# A common regulatory haplotype doubles lactoferrin concentration in milk

**DOI:** 10.1186/s12711-024-00890-x

**Published:** 2024-03-28

**Authors:** Thomas J. Lopdell, Alexander J. Trevarton, Janelle Moody, Claire Prowse-Wilkins, Sarah Knowles, Kathryn Tiplady, Amanda J. Chamberlain, Michael E. Goddard, Richard J. Spelman, Klaus Lehnert, Russell G. Snell, Stephen R. Davis, Mathew D. Littlejohn

**Affiliations:** 1 Research & Development, Livestock Improvement Corporation, Ruakura Road, Hamilton, New Zealand; 2 School of Biological Sciences, University of Auckland, Private Bag 92019, Auckland, New Zealand; 3 Agriculture Victoria, AgriBio, Centre for AgriBiosciences, Bundoora, VIC Australia; 4 Faculty of Veterinarian and Agricultural Science, The University of Melbourne, Parkville, VIC Australia; 5 Auckland War Memorial Museum, Victoria Street West, Auckland, New Zealand; 6 AL Rae Centre for Genetics and Breeding, Massey University, Palmerston North, New Zealand

## Abstract

**Background:**

Bovine lactoferrin (Lf) is an iron absorbing whey protein with antibacterial, antiviral, and antifungal activity. Lactoferrin is economically valuable and has an extremely variable concentration in milk, partly driven by environmental influences such as milking frequency, involution, or mastitis. A significant genetic influence has also been previously observed to regulate lactoferrin content in milk. Here, we conducted genetic mapping of lactoferrin protein concentration in conjunction with RNA-seq, ChIP-seq, and ATAC-seq data to pinpoint candidate causative variants that regulate lactoferrin concentrations in milk.

**Results:**

We identified a highly-significant lactoferrin protein quantitative trait locus (pQTL), as well as a *cis* *lactotransferrin* (*LTF*) expression QTL (*cis*-eQTL) mapping to the *LTF* locus. Using ChIP-seq and ATAC-seq datasets representing lactating mammary tissue samples, we also report a number of regions where the openness of chromatin is under genetic influence. Several of these also show highly significant QTL with genetic signatures similar to those highlighted through pQTL and eQTL analysis. By performing correlation analysis between these QTL, we revealed an ATAC-seq peak in the putative promotor region of *LTF*, that highlights a set of 115 high-frequency variants that are potentially responsible for these effects. One of the 115 variants (rs110000337), which maps within the ATAC-seq peak, was predicted to alter binding sites of transcription factors known to be involved in lactation-related pathways.

**Conclusions:**

Here, we report a regulatory haplotype of 115 variants with conspicuously large impacts on milk lactoferrin concentration. These findings could enable the selection of animals for high-producing specialist herds.

**Supplementary Information:**

The online version contains supplementary material available at 10.1186/s12711-024-00890-x.

## Background

Milk production and composition are very important to farmers, dairy processing companies, and consumers. Accordingly, there has been a considerable amount of research into the genetic basis underlying phenotypic variation in milk. While numerous genome-wide association studies (GWAS) have surveyed the abundance of major milk components [1–4], genetic analysis of minor milk components is less understood. The globular glycoprotein lactoferrin (Lf), encoded by the *lactotransferrin* gene (*LTF*), forms a minor but economically valuable component of the whey fraction of milk protein. Lf has a high binding affinity for Fe ^3+^, and forms a component of the non-specific immune system [5], exhibiting broad antibacterial, antifungal and antiviral activity [6]. This anti-microbial effect confers passive immunity to the neonate mammal until its own immune system has matured. The bacteriostatic and bactericidal abilities of Lf arise from the ability of the protein to sequester iron [7], thereby reducing its bioavailability to bacteria [8]. These effects also derive from the direct binding of a peptide formed from the N-terminus section of the protein (known as lactoferricin) to the cell membranes of a wide range of pathogenic species [9]. Lf is also active against a number of viruses [7], primarily those with membrane envelopes, including influenza virus [10, 11], hepatitis C virus [12], and potentially SARS-CoV-2 [13–15].

Lactoferrin concentrations are highly variable in milk, varying by over an order of magnitude [16] between individuals. Although much of this variation can be explained by factors such as the stage of lactation and infection in the mammary gland (mastitis), a proportion of the variance appears to be under genetic control [17, 18]. Due to its many potential pharmaceutical uses, it may be beneficial to select for cattle with a genetic propensity for producing higher quantities of Lf. To this end, our aim was to investigate the genetic control underpinning lactoferrin production, using molecular phenotypes to help identify candidate genetic variants potentially responsible for these effects.

## Methods

### Animal populations

This study used two overlapping sets of animals which had been phenotyped at different dates as part of other large experiments. This section describes the breed and other characteristics of these populations. Phenotypic and genomic data descriptions follow in the subsequent sections.

The first population was measured for milk Lf concentration as part of a Holstein-Friesian $\times$ Jersey crossbreeding (FJX) trial [19, 20]. This trial was conducted using an F2 trial design with a half-sibling family structure, where reciprocal crosses between Holstein-Friesian and Jersey animals were carried out to produce six F1 bulls. A herd of F2 cows was subsequently produced by mating the F1 bulls with F1 cows over two seasons (born spring 2000 and spring 2001). In total, 724 F2 cows entered their second lactations (spring 2003 and spring 2004), of which 706 were sampled at least once to determine milk Lf concentrations (see Lf quantification methodology section below). All animals were raised using a seasonal pasture-based management system as typically used in New Zealand, under a twice-daily milking regime.

The second population of 411 animals was sampled to generate a previously published [3, 21] RNA-seq dataset. After sample quality control (see methods in [3]), 372 mixed-breed, mixed age animals of Holstein-Friesian and Jersey ancestry were retained. A subset of 22 animals were F2 animals from the FJX population. The remaining 350 animals were primarily of Holstein-Friesian ancestry, comprising 211 pure-bred Holstein-Friesians, three pure-bred Jerseys, and 136 cross-bred animals, where pure-bred is defined as $\ge 14/16$ths ancestry based on pedigree information. A subset of 99 animals from the 372 animal RNA-seq population was also analysed using chromatin immuno-precipitation with sequencing (ChIP-seq; see the methodological description below). An additional non-overlapping subset of 199 animals was analysed using the assay for transposase-accessible chromatin with sequencing (ATAC-seq) to identify windows of open chromatin.

### Lf protein and RNA expression quantification

Lactoferrin protein concentration was measured in the FJX population at up to three time points during the second lactation of each animal: at peak lactation (35 days post-calving; N = 621), mid-lactation (mid-November; N = 648), and late lactation (late February; N = 611). On each test day, samples were collected (combined across all four quarters) during both the a.m. and p.m. milkings, then combined to yield a single composite sample for each animal. Lf concentrations were measured using reversed-phase high-performance liquid chromatography (RP-HPLC) as previously described [22, 23]. These measurements were aggregated and adjusted using a repeated-measures model in ASReml-R [24] for 706 animals with at least one Lf record. Fixed effects were fitted for the sample collection period (early, mid, or late), Friesian breed proportion (in contrast to Holstein; the Jersey proportion was always 50% as all animals were F2 crosses of Holstein-Friesian $\times$ Jersey), and breed heterosis, with random effects for animal (using a relationship matrix defined by the recorded pedigree) and a permanent environmental effect also fitted. The aggregate phenotype was calculated for each animal as the sum of the additive animal component and the mean of the residual components for that animal’s measurements.

Sample skewness estimates for the four Lf phenotypes (three collection periods plus aggregate) were calculated using Eq. (1), where *n* is the number of samples, *s* is the sample standard deviation, and *x* is each Lf record across the samples. As the estimates obtained indicated strong positive skews in Lf concentrations (see results section), additional phenotype values were created for which the values were log-transformed. A log version of the aggregate phenotype was not generated, because the values for the aggregate phenotype are centred on zero, and therefore feature a large proportion of negative values.

1 $$\begin{aligned} b_1 = \frac{ \sum ^n_{i=1}{\left( x_i - \bar{x} \right) ^3} }{n \cdot s^3}. \end{aligned}$$

For quantification of RNA expression, biopsies of lactating mammary tissue taken from the RNA-seq population animals were sequenced using the Illumina HiSeq 2000 platform as described previously [3, 21]. Paired-end RNA reads were processed using the Trimmomatic software package (version 0.39) [25] with settings LEADING:20 TRAILING:20 SLIDINGWINDOW:3:15 MINLEN:50, then mapped to a masked version of the ARS-UCD1.2 bovine reference genome (including a Y chromosome), where known variants were replaced by bases that matched neither of the two alleles (to avoid mapping bias; see [26] for details) using STAR (version 2.7.0) [27] in two passes. In the first pass, the STAR index file was generated using junctions extracted from the NCBI assembly GCF_002263795.1 with annotation release 106. Novel splice junctions with at least five uniquely mapping supporting reads were extracted, and used to augment the annotation set used for the second pass.

To produce a gene expression phenotype for use in the expression quantitative trait locus (eQTL) association analysis (see below), reads mapping to the *LTF* gene were counted for each animal using the featureCounts function of the Subread package (version 1.5.3) [28]. Read counts were subsequently normalised using the variance-stabilising transformation (VST) implemented in the DESeq2 (version 1.26) R package. Outlier samples were detected using principal component analysis (PCA) on the VST-transformed counts, where any samples $>4$ standard deviations from the mean, in any of the first six principal components(PC), were excluded.

### ChIP-seq analysis and bioinformatics

The ChIP-seq data used here were generated as part of a previous study [26]. Briefly, chromatin immunoprecipitation was performed using the Magnify Chromatin Immunoprecipitation kit (Thermofisher) for three histone modifications: histone 3 lysine 4 mono- and trimethylation (H3K4Me1 and H3K4Me3), and lysine 27 acetylation (H3K27ac). Libraries were produced for 99 samples for the H3K4Me1 and H3K4Me3 modifications, and 37 samples for H3K27ac. Each library was sequenced to a depth of 20–200 million reads, and reads were trimmed using the Trimmomatic software package (version 0.39) [25] as for the RNA-seq data described above. Reads were mapped to the masked reference genome described above, using BWA-MEM version 0.7.17-r1188 [29], with poor quality and duplicate reads being removed using the Samtools package (version 1.9) [30].

Consensus ChIP peaks were called by randomly downsampling equal numbers of reads from each BAM file, followed by merging into a consensus BAM file for each of the three histone modifications (both steps were performed using SAMtools version 1.9). Consensus ChIP peaks were called on the consensus BAM file using MACS2 version 2.1.1 [31], with low-depth WGS prepared from the same samples (input reads) as a control. Peak calling was performed using the broadPeak algorithm for the H3K4Me1 data, and the narrowPeak algorithm for the remainder. This yielded 971 H3K27ac peaks within 1 Mb of the *LTF* gene, along with 429 for H3K4Me1 and 782 for H3K4Me3 within the window on *Bos taurus* chromosome (BTA) 22:51946110–53986647 (see Additional file 1: Tables S1–S3 for peak caller outputs).

Reads under each consensus peak were counted using the featureCounts function of the Subread software package (version 1.5.3) [28], for both the ChIP-seq peak data and the corresponding input data. As a quality control step, a length-adjusted read count was produced for each peak by dividing the read count by the number of bases covered by the peak. Peaks with an adjusted read count below the first percentile of all peaks were removed. Peaks were also removed when the adjusted input read count was over $5\times$ the average across all reads, to remove potential false positive peaks caused by artefacts in the reference genome. This yielded a filtered data set of 793, 353, and 725 peaks within 1 Mb of *LTF* for H3K27ac, H3K4Me1, and H3K4Me3, respectively.

2 $$\begin{aligned} {\textbf {t}}_{i} = \sum _{j=1}^{p} {\textbf {C}}_{i,j}, \qquad {\textbf {f}}_{i} = \frac{{\textbf {t}}_{i}}{\text {mean}({\textbf {t}})}, \qquad {\textbf {C}}^N_{i,j} = \frac{{\textbf {C}}_{i,j}}{{\textbf {f}}_{j}}. \end{aligned}$$

To enable the identification of histone accessibility QTL (hQTL) that map near the *LTF* gene, phenotypes for chromatin openness were developed, with the aim of being suitable for mixed model analyses. The read counts for both the ChIP-seq (${\textbf {C}}_{p\times {}n}$) and input (${\textbf {I}}_{p\times {}n}$) data were stored as matrices, where each row represented a ChIP-seq peak, each column represented an animal. Vectors of total read counts per animal (${\textbf {t}}$) were calculated by summing the counts in each column of the count and input matrices (example for the ChIP-seq counts in Eq. (2); calculations proceed analogously for the input read counts). The read depth normalisation factors (${\textbf {f}}$) for each animal were then calculated, representing the total count for each animal divided by the mean across all animals; this yielded a number representing the read depth of each animal relative to the average animal. The read counts are then divided by the read depth factors to yield the adjusted count matrices (${\textbf {C}}^N$ and ${\textbf {I}}^N$ for the Peak and Input counts, respectively). Using the vectors ${\textbf {y}}_i = \log _e( {\textbf {C}}^N_{i,\cdot } + 1 )$ and ${\textbf {x}}_i = \log _e( {\textbf {I}}^N_{i,\cdot } + 1 )$, the linear model ${\textbf {y}}_i = a + b\cdot {\textbf {x}}_i + {\textbf {e}}_i$ was fitted using ordinary least squares for each peak (*i*) across all animals, with the residuals (${\textbf {e}}_i$) yielding the final phenotype used for hQTL discovery.

As a final step, further filtering was applied to remove samples with anomalous read cover; for example, samples prepared from an incorrect tissue, or which had issues with library preparation. PCA was conducted to identify outlier samples following a method similar to that of Ellis et al [32], and any sample falling more than four standard deviations from the mean in any of the first seven principal components was excluded. This resulted in a final dataset containing 34, 94, and 95 genotyped animals for H3K27ac, H3K4Me1, and H3K4Me3, respectively.

### Measuring chromatin accessibility using ATAC-seq

A subsample of 199 mammary tissue secondary biopsies (collected as part of the RNA-seq experiment) were assayed for open chromatin regions using the Assay for Transposase Accessible Chromatin using Sequencing (ATAC-seq) method [33]. Libraries were prepared using the commercial ATAC-Seq Kit provided by Active Motif (Carlsbad, CA, USA), then sequenced using the Novaseq 6000 genome analyser (Illumina Inc, CA, USA), targeting 25 million 150 bp paired-end reads per sample. Read processing, mapping, and counting proceeded as described above for the ChIP-seq analysis. Peak calling was performed using the MACS3 software package (version 3.0.0a7) within 1 Mb of the *LTF* gene, on a consensus BAM file produced by sampling 5% of the reads from each individual sample BAM file. This yielded 207 peaks within the window BTA22:51946110–53986647 (see Additional file 1: Table S4).

Phenotypes for chromatin accessibility QTL (caQTL) discovery were produced in a similar fashion to the ChIP-seq phenotypes, with the exception that, because individual input samples were not available, a consensus input BAM file was created by sampling 1% of the reads from each of the ChIP-seq input files. While this method would not identify any problematic regions specific to an individual animal, it will enable the identification of regions of the genome that cause read pile-ups due to inherent reference assembly problems. One sample was identified as an outlier via PCA (as described above) and removed from the QTL discovery set, resulting in a QTL discovery population of 193 animals after excluding animals that failed genotype concordance.

### Genotyping and imputation

Within the 706 animals in the FJX population, 679 were genotyped using the Illumina Bovine50k (50k) chip panel. Of these, 12 have also been re-genotyped on the high-density Illumina BovineHD 777k (HD) chip panel, with the remainder imputed to this panel using Beagle version 5.0 [34]. For the RNA-seq population of 372 animals, 350 animals were genotyped on the HD panel. The remaining 22 (overlapping the FJX population) were genotyped using the Illumina Bovine50k panel, then imputed to the HD panel using Beagle (version 5.0). The subset of 99 animals used for the ChIP-seq experiment were all genotyped on the HD panel, as were the 199 animals used for the ATAC-seq experiment. Variants (both imputed and genotyped) were subsequently imputed to whole-genome sequence resolution using Beagle 5.0 across a window encompassing 1 Mb of sequence either side of the annotated *LTF* gene (BTA22:51946110–53986647). This window contained 608 HD markers, which were imputed up to a total of 43059 whole-genome sequence (WGS) markers using a mixed-breed reference population of 1300 sequenced cattle, comprising 231 Jerseys, 392 Holstein-Friesians, and cross-bred animals, and forming a superset of our previously published imputation reference population of 556 animals [21]. After filtering for allelic dosage R^2^ (DR2) $>0.9$ and minor allele frequency (MAF) $>0.005$, a final set of 11,736 WGS variants was produced.

Separately, variants were imputed over the same genomic window surrounding the gene as described above for the RNA-seq population, producing a final set of 10,916 imputed WGS markers. This set is slightly smaller than the set selected for protein QTL discovery, as more low-frequency markers were dropped because insufficient numbers of observations were available for their alternative alleles, as a result of the smaller number of samples in this dataset.

### Association analyses for QTL discovery

Marker-based heritability estimates for Lf concentrations were calculated using the GCTA software package (version 1.93.2) [35] with the restricted maximum likelihood average information (REML-AI) method. A genomic relationship matrix (GRM) was produced using GCTA with the actual (where available) and imputed HD genotypes. Genome-wide association studies (GWAS) were conducted for Lf protein at three sampling periods (peak, mid, and late lactation), plus the aggregated phenotype produced using the repeated-measures model, as described above. GWAS was conducted using the GCTA software package (version 1.93.2) with the same HD genotypes and GRM, along with a covariate for animal birth year. To fine-map the region 1 Mb either side of the *LTF* gene, an additional analysis was undertaken for each phenotype using an imputed WGS-resolution variant set, comprising 11,736 variants that mapped to this genomic window after removing variants with MAF less than 0.005 and imputation DR2 less than 0.9. This analysis was carried out as per the HD variant set, except that the 598 HD variants that mapped within the target window were excluded from the GRM calculation, yielding a leave-one-segment-out (LOSO) design.

Gene eQTL analysis for the *LTF* gene was performed similarly using the VST-transformed phenotype described above. The analysis was run using GCTA version 1.93.2 [35] and a GRM calculated for the RNA-seq population of animals with physically genotyped HD genotypes. The mixed linear model analysis (MLMA) method in GCTA was run using a whole genome HD genotype set with leave-one-chromosome-out (LOCO), to identify any *LTF* *trans*-eQTL. Fine mapping of the *cis*-eQTL was undertaken using 10916 imputed sequence variants with filtering as described above.

Histone QTL (hQTL) and caQTL were identified similarly using the chromatin openness phenotypes described above, by applying a GCTA MLMA-LOCO model with imputed sequence variants mapping within 1 Mb of each ChIP-seq or ATAC-seq peak within 1 Mb of the *LTF* locus. This yielded a mean of 10136 variants per peak analysed. As the ChIP-seq and ATAC-seq samples formed a subset of the RNA-seq samples, the same GRM was reused for the chromatin QTL analyses.

As a final step, the Pearson and Spearman correlations between Lf pQTL and the *LTF* eQTL were calculated using the R software package [36]. Correlations were calculated using both the $\beta$ allele effects and the $-log_{10}(p)$-values for each variant within 50 kb of the *LTF* gene, i.e., between positions 52896110 and 53036647 on BTA22. Similarly, correlations were calculated for the *LTF* eQTL with each of the hQTL and caQTL for which the corresponding ChIP-seq or ATAC-seq peak lay within 1 Mb of *LTF*. To summarise the linear relationships between pQTL, eQTL, and hQTL/caQTL simultaneously, we then performed a principal components analysis (PCA) for each triplet of pQTL, eQTL, and hQTL/caQTL, then determined the percentage of variance for each that could be explained by the first principal component. This approximates a three-dimensional analogue to the R^2^ (called a pseudo-R^2^ in this text).

### Identification of candidate transcription factor binding sites (TFBSs)

Sequences within open chromatin regions with significant hQTL were examined to predict the positions of transcription factor binding sites (TFBSs). Significant hQTL for ATAC-seq ($n=32$) and ChIP-seq ($n=4$ and 3 for H3K4Me1 and H3K4Me3 respectively) were selected, where at least one hQTL variant was observed with $p<{5 \times 10^{-8}}$. The reference DNA sequence under each peak was extracted in FASTA format and loaded into R version 4.1 using the Bioconducter package Biostrings (version 2.62). Position weighted matrices (PWMs) representing transcription factor binding motifs were loaded from the JASPAR database using the package JASPAR2020 (version 0.99.10) for the CORE collection defined in that package (comprising profiles representing curated, non-redundant binding site sequences), with the taxonomic group ‘vertebrates’ ($n=746$ PWMs), and also from the POLII collection ($n=13$ PWMs), comprising sequences for RNA polymerase II promoter elements. Candidate TFBSs were then located on the DNA sequences using the PWMs with the package TFBSTools (version 1.32), and filtered to keep only those with a $\text {relScore} \ge 0.9$.

## Results

### Milk Lf concentrations and genetic correlations

Milk Lf concentrations were measured in 706 animals at up to three time periods each during a single milking season (peak lactation, mid lactation, and late lactation) using HPLC. Lf concentrations were highly variable, with a difference of about two orders of magnitude between the highest and lowest concentrations in each time period (see Additional file 2: Fig. S1). In addition, an aggregate phenotype was produced by running a repeated-measures model in AS-REML [24]. Summary statistics for all four phenotypes are in Table 1. The lowest Lf concentrations were observed at peak lactation, with the highest values seen at mid-lactation, followed by a modest reduction in late lactation. Table 1 also shows the narrow-sense SNP heritabilities ($h^2_{\text {SNP}}$) calculated for each phenotype. In general, values of 0.3 to 0.4 were observed (with the exception of mid lactation), suggesting that Lf concentration is moderately heritable. All heritability estimates were statistically significant by the likelihood ratio test (maximum p-value ${1.77 \times 10^{-4}}$). The highest heritability estimate was observed with the aggregate repeated-measures phenotype (0.433), followed by LogPeak (0.416) and LogLate (0.413).

### Association mapping of protein QTL (pQTL)

Performing a GWAS using HD genotypes for each of the four Lf phenotypes (peak, mid, late, and aggregated) yielded significant ($p<\frac{0.05}{631896}={7.9 \times 10^{-8}}$) QTL in all cases: minimum p-values attained were ${1.54 \times 10^{-14}}$, ${1.73 \times 10^{-10}}$, ${2.27 \times 10^{-23}}$, and ${3.09 \times 10^{-24}}$ for the peak, mid, late, and aggregate phenotype, respectively. With the exception of the late lactation phenotype, the most significant SNP identified was rs110659162 on BTA22 at position 52986092, located within intron 16 of the *LTF* gene (referencing Ensembl transcript ENSBTAT00000001704.5). For the late lactation phenotype, the most significant variant was rs109183581 on BTA22 at position 52954126 in the first intron of *LTF*.

Compared to the non-log phenotypes, the log phenotypes gave stronger genetic signals. The most significant variant for the log-peak lactation phenotype (LogPeak) was rs109183581 with a p-value of ${4.01 \times 10^{-23}}$, and for the log-mid lactation phenotype (LogMid), the most significant variant was the synonymous variant rs43765460 at position 52969419, (*LTF* exon 9 of 17) with a p-value of ${2.89 \times 10^{-14}}$. The log-late lactation phenotype (LogLate) yielded a haplotype comprising seven SNPs (minimum pairwise linkage disequilibrium (LD): R^2^ = 0.998) within the window 52940222–52951641 on BTA22, all with a p-value of ${8.05 \times 10^{-30}}$. Four of these variants were predicted by Ensembl’s Variant Effect Predictor (VEP) to be intergenic; however, the remaining three (rs109348197, rs134043953, and rs137054020) map between 0.9–4.5 kb upstream of *LTF*, approximately where promoter elements could be expected to be located. Manhattan plots for the three log-phenotypes, as well as the Aggregate phenotype, are shown in Additional file 3: Fig. S2. Strikingly, the estimates of the effect of this pQTL in *cis* were substantial, with a more than two-fold difference between opposing homozygotes.

**Fig. 1:**
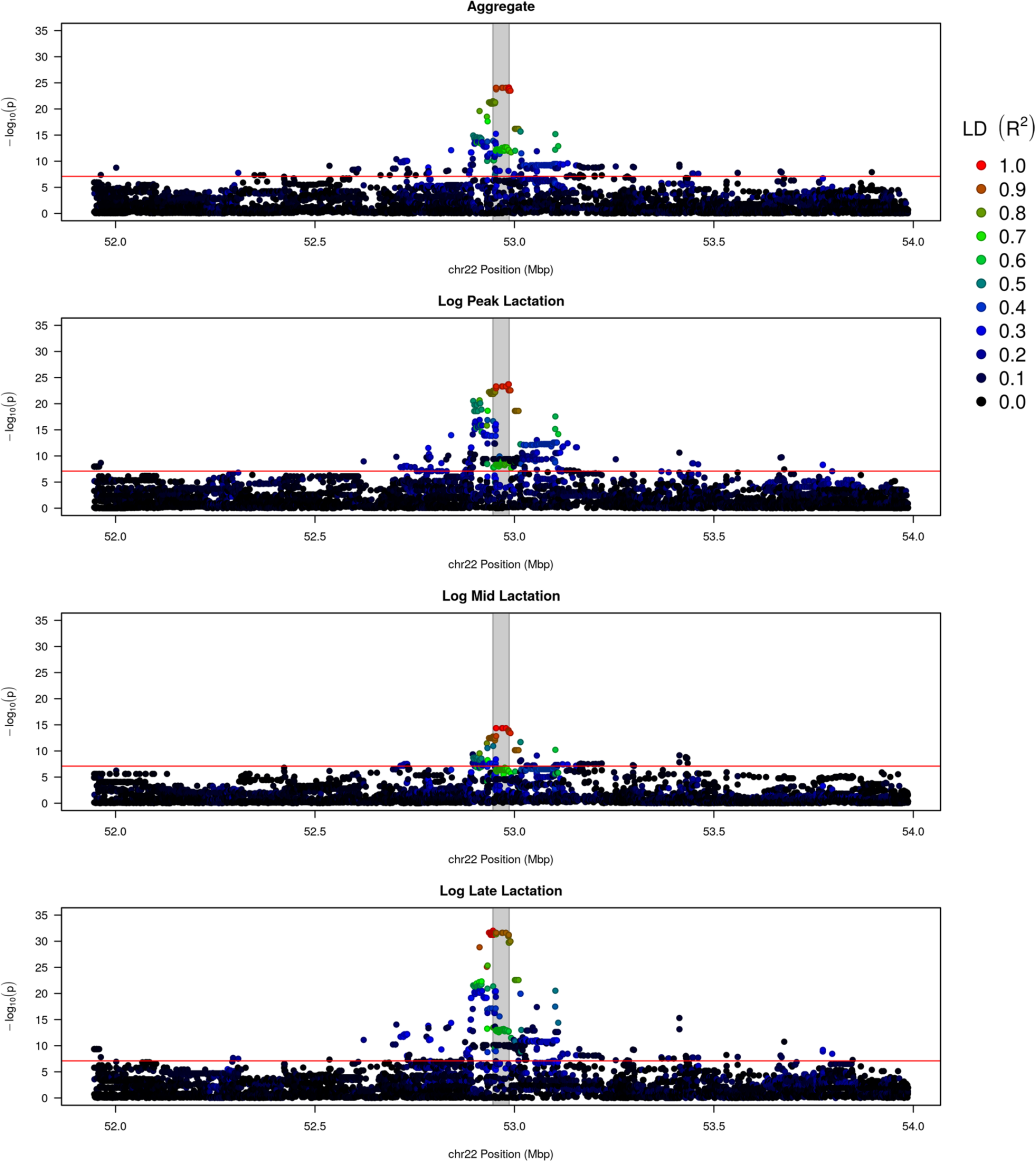
Fine-mapping of the Aggregate, LogPeak, LogMid, and LogLate phenotypes, within the 2 - Mb window surrounding the *LTF* gene on BTA22. Colours indicate linkage disequilibrium (R^2^) between each variant and the variant with the smallest p-value. The grey band indicates the location of the *LTF* gene (5′ on the left)

To fine map the Aggregate and Log pQTL located at the *LTF* locus, we defined a genomic window of interest comprising 1 Mb on each side of the *LTF* gene using an imputed WGS resolution variant set. The most significantly associated variants ($p={1.93 \times 10^{-24}}$) in peak lactation were rs133536129 (BTA22:52984449; intron 15) and rs211296757 (BTA22:52985300; intron 16). The LogMid phenotype yielded a haplotype of eight variants (all with $p={4.30 \times 10^{-15}}$), of which seven were intronic (introns 1, 7, 9, and 13), and the eighth was the same synonymous variant (rs43765460; exon 9) identified in the HD GWAS described above. The top variant for the LogLate phenotype, rs137774559 (BTA22:52946182), was predicted to be intergenic, with a p-value of ${9.50\times 10^{-33}}$, and the top variant for the Aggregate phenotype was rs109183581, the same variant as seen for the late-lactation phenotype above, with a p-value of ${1.23 \times 10^{-8}}$. These variants were all highly correlated in the GWAS population, giving pairwise R^2^ values ranging from 0.84 to 1.00, with rs109183581 showing the lowest R^2^ with the remaining variants (from 0.81 to 0.88). Manhattan plots, coloured by LD with the most significant variant in each analysis, are in Fig. 1. See Additional file 1: Tables S5–S8 for full pQTL results.

All the tag variants for the Log phenotypes, i.e., the most significant variants in the QTL, showed surprisingly large Lf protein effects, especially considering that these variants were very common in the study population (MAF of around 0.45). The largest effect was observed for LogPeak, $0.56 \pm 0.055$ on a $\log _e$-scale, equivalent to a $1.75\times$ higher milk Lf concentration per allele ($3.08\times$ between homozygotes) on a linear scale. Mean Lf concentrations (${\hbox {mgL}}^{-1}$) observed for peak lactation were 32.2, 69.3, and 111.5 for the GG, GT, and TT genotypes of rs133536129, respectively. A similarly large effect was observed for the LogLate phenotype, with a $\log _e$-scale effect of $0.50 \pm 0.042$, equivalent to $1.65\times$ on a linear scale, or $2.74\times$ between homozygotes. Mean Lf concentrations for late lactation were 71.5, 159.8, and 221.9 ${\hbox {mgL}}^{-1}$ for the TT, CT, and CC alleles of rs137774559, respectively.

The population frequency of rs137774559, the top associated variant for LogLate, was also determined in the data set from a larger, previously reported population [37] of 38085 mixed-breed cows (UMD3.1 position BTA22:53514853). Within the total population, the minor allele was ‘T’, with a MAF of 0.4325. We also determined the allele frequency in pure-bred subpopulations of this larger population, with Holstein-Friesians (n = 8504) showing a MAF of 0.3332 for the same allele, and Jerseys (n = 4804) having the opposite minor allele ‘C’, with a MAF of 0.3851. The remaining animals comprised a mix of predominantly Holstein $\times$ Jersey crosses, and other minor pure and mixed breeds. Since the allele associated with the highest concentrations of Lf was the ‘C’ allele, this implies that Holstein-Friesian cattle are in general genetically predisposed to higher Lf levels in milk than Jersey cows, although this contradicts previous research showing that Jersey milk has significantly higher Lf concentrations than Holstein milk [38]. Unfortunately, breed-specific analyses could not be conducted in the current study, as all the cows for which Lf concentration data were available were Holstein $\times$ Jersey crosses.

### Investigation of candidate causative regulatory variants for the Lf pQTL

Several small studies have looked for genetic effects on milk Lf concentration, typically examining only one or two promoter or 5′-UTR variants, many of which were captured in the current dataset. To verify whether or not these variants represented QTL that differed from that presented here, the pQTL analysis was repeated with the genotype of rs133536129 (the top variant for LogPeak) fitted as a covariate. The promoter variant “Lf-962” was previously shown to be weakly associated with milk Lf concentration [39]. In the current study, this variant yields p-values of ${2.32 \times 10^{-16}}$ and ${4.65 \times 10^{-21}}$ for peak and late lactation respectively, compared to ${1.93 \times 10^{-24}}$ and ${9.50 \times 10^{-33}}$ for the lead variants of each trait. The Lf-962 effect becomes non-significant after fitting rs133536129, with p-values of 0.177 and 0.188, although the LD between the two markers was weak (R^2^ = 0.273).

A second promoter variant “Lf-28” (rs41256920) has also been associated with milk Lf concentration [40]. This variant is in moderately strong LD with rs133536129 (R^2^ = 0.701), and is associated with both peak and late lactation Lf concentrations in the current study ($p={4.67 \times 10^{-9}}$ and ${1.25 \times 10^{-13}}$, respectively). As above, this variant was noticably less significant than the lead variants presented in the current study, and it also becomes non-significant after fitting rs133536129 ($p={0.159}$ and 0.830). A third variant, in the 5′-UTR, that has previously been associated with Lf concentrations is “Lf+32” (rs43706485) [39, 41]. Although this variant is in moderately weak LD with rs133536129 (R^2^ = 0.327), fitting the latter as a covariate results in the moderately significant p-values for Lf+32 becoming non-significant: from ${8.08 \times 10^{-17}}$ to 0.325 for LogPeak, and from ${4.54 \times 10^{-20}}$ to 0.679 for LogLate.

Conversely, fitting the genotype of Lf-926 resulted in the p-value of rs133536129 remaining significant, although it decreased to ${2.14 \times 10^{-6}}$, suggesting that Lf-926 captures only part of the genetic signal at this locus. Similar results were observed after fitting Lf-28 (rs133536129 $p={5.50 \times 10^{-11}}$) or Lf+32 ($p={2.94 \times 10^{-5}}$). Collectively, these observations suggest that these promoter-region variants capture a portion of the signal for the same LogPeak and LogLate pQTL as identified in this study, but also show that this signal is better represented by rs133536129 and other variants within the core haplotype.

### *LTF* expression and identification of eQTL

RNA-seq data from lactating mammary tissue confirmed that *LTF* is moderately highly expressed (median transcripts per million (TPM) = 350.01, mean = 597.34) in all animals (minimum TPM = 28.44). Similar to the protein phenotypes, *LTF* expression showed a strong positive skew, with a sample skewness estimate of 4.946. However, data transformation using the VST [42] reduced the skew of the expression phenotype to minimal levels (sample skewness = 0.242). Therefore, we can anticipate that skewness is unlikely to lead to any false-positive results in eQTL discovery.

A GWAS was conducted using the VST expression phenotype with the HD genotype set to identify eQTL for the *LTF* gene. A highly significant *cis*-eQTL was identified (minimum p-value: ${1.38 \times 10^{-33}}$) on BTA22 at 52.95 Mbp, overlapping the transcription start site (TSS) of the gene (at position 52946110). The three most significantly associated markers (rs134043953, rs137054020, and rs42013171) formed a haplotype with a MAF of 0.37 (minimum pairwise R$^{2}>0.99$) overlapping the top HD-chip variants identified for the LogLate phenotype. No marker outside BTA22 was significantly associated with *LTF* transcript levels (all $p>{7.9 \times 10^{-8}}$). Fine mapping of the locus using imputed sequence data gave a minimum p-value of ${1.50 \times 10^{-32}}$ for marker rs800016664 at position 52941483, 4.6 kb upstream of *LTF* (see Additional file 1: Table S9 for full *LTF* results). With the same dataset and model, the top HD variants yielded p-values of ${2.81 \times 10^{-32}}$, suggesting that the causal haplotype was adequately tagged by the HD-chip platform.

### Histone modification and chromatin accessibility peaks identified

In total, 2182 ChIP-seq peaks were identified within 1 Mb of the *LTF* gene, comprising 971 peaks for H3K27ac, 429 for H3K4Me1, and 782 for H3K4Me3. The five most significant peaks for each of these histone modifications, as ranked by Q-value, are listed in Table 2. A number of peaks were detected that mapped to loci overlapping the *LTF* gene, including 20 for H3K27ac, nine for H3K4Me1, and 17 for H3K4Me3. In all cases, the peak with the highest score overlapped the annotated *LTF* TSS (BTA22:52952571): 52951712–52956226 for H3K27ac (MACS2 score 1665), 52950247–52958565 for H3K4Me1 (133), and 52951824–52956227 for H3K4Me3 (9522). Although these scores are noticeably lower than others in the wider region, they still represent highly-significant peaks. Within the window used for association analyses (i.e., within 1 Mb of *LTF*; BTA22:51946110–53986647), 971, 429, and 782 peaks were detected for the H3K27ac, H3K4Me1, and H3K4Me3 histone modifications, respectively (See Additional file 1: Tables S10–S12 for summary hQTL results), covering 33.2%, 47.0%, and 19.2% of the bases in the window. The average lengths of the peaks were 698.0 bp, 2237.5 bp, and 499.8 bp, respectively. At least one of the top-ranking trait associated SNPs for each of the fine-mapped pQTL fell within a ChIP-seq peak (Table 3).

In total, 207 ATAC-seq peaks were detected within the interval encompassing 1 Mb on each side of *LTF* (see Additional file 1: Table S13 for caQTL summary results). The five most significant ATAC-seq peaks are listed in Table 2. The average length of the 207 peaks was 723.5 bp (SD = 426.0), collectively covering 7.3% of the genomic window. One ATAC-seq chromatin accessibility peak overlapped the 5′-UTR and first exon of the *LTF* gene (peak ATAC-94 at 52953062–52954626, Qval = 443.6). Although this peak did not contain the currently annotated TSS, it did overlap the non-canonical TATA box identified by Zheng et al. [43]. An additional four ATAC-seq peaks were located within 20 kb upstream of *LTF*, the most significant of which (peak ATAC-90) mapped to 52934507–52936357 with a Qvalue of 796.31. All four of the ATAC-seq peaks overlapped with ChIP-seq peaks for all three histone modifications.

### Chromatin QTL

Using a threshold of $p<{7.9 \times 10^{-8}}$, seven ChIP-seq peaks exhibited significant hQTL, of which four were observed for the H3K4Me1 histone modification, with the remaining three for the H3K4Me3 histone modification. No H3K27ac peaks yielded significant QTL at this threshold, which is likely due to the smaller sample size available for this histone modification. Among the four H3K4Me1 peaks with significant QTL, one mapped upstream of the *SCAP* gene (peak Me1-65), two were within the *LTF* gene (peaks Me1-196 and Me1-202), and one overlapped the TSS of the *SACM1L* gene (peak Me1-343). Among the three peaks identified with significant QTL for the H3K4Me3 marker, one mapped upstream of the *SCAP-202* transcript (peak Me3-61), one overlapped with the TSS of the *LTF* gene (peak Me3-391), and one was within the *LARS2* gene (peak Me3-737). Adopting a less stringent p-value threshold of $p<{1 \times 10^{-5}}$ resulted in one significant QTL for an H3K27ac peak (peak Ac-886), 17 for H3K4Me1 and nine for H3K4Me3. The H3K27ac peak mapped to an intron of the *LARS2* gene.

Compared to the ChIP-seq peaks, QTL were more frequently observed for the ATAC-seq peaks, which is likely due to the larger sample size, with 32 peaks exhibiting a significant caQTL at $p<{7.9 \times 10^{-8}}$, and 50 peaks exhibiting a significant caQTL at $p<{1 \times 10^{-5}}$. The ATAC-seq peaks were also distributed across many more genes. However, one (peak ATAC-94) was located close to the TSS of the *LTF* gene. An additional three peaks (ATAC-91, ATAC-92, and ATAC-93) mapped within 15 kb upstream, and another overlapped exon 15 (peak ATAC-102). Further away from the *LTF* gene, another peak (ATAC-71) exhibiting a significant QTL overlapped the TSS of the *ALS2CL* gene, with two additional peaks (ATAC-73 and ATAC-75) mapping within the gene. Other peaks with significant caQTL mapped within the genes *SMARCC1* (peak ATAC-4), *CCDC12* (peaks ATAC-51 and ATAC-53), *LRRC2* (ATAC-88), *FYCO1* (ATAC-139), *LIMD1* (ATAC-167, ATAC-169, and ATAC-174), and *LARS2* (ATAC-184, ATAC-184, ATAC-185, ATAC-192, and ATAC-193).

### Co-regulation between QTL

**Fig. 2:**
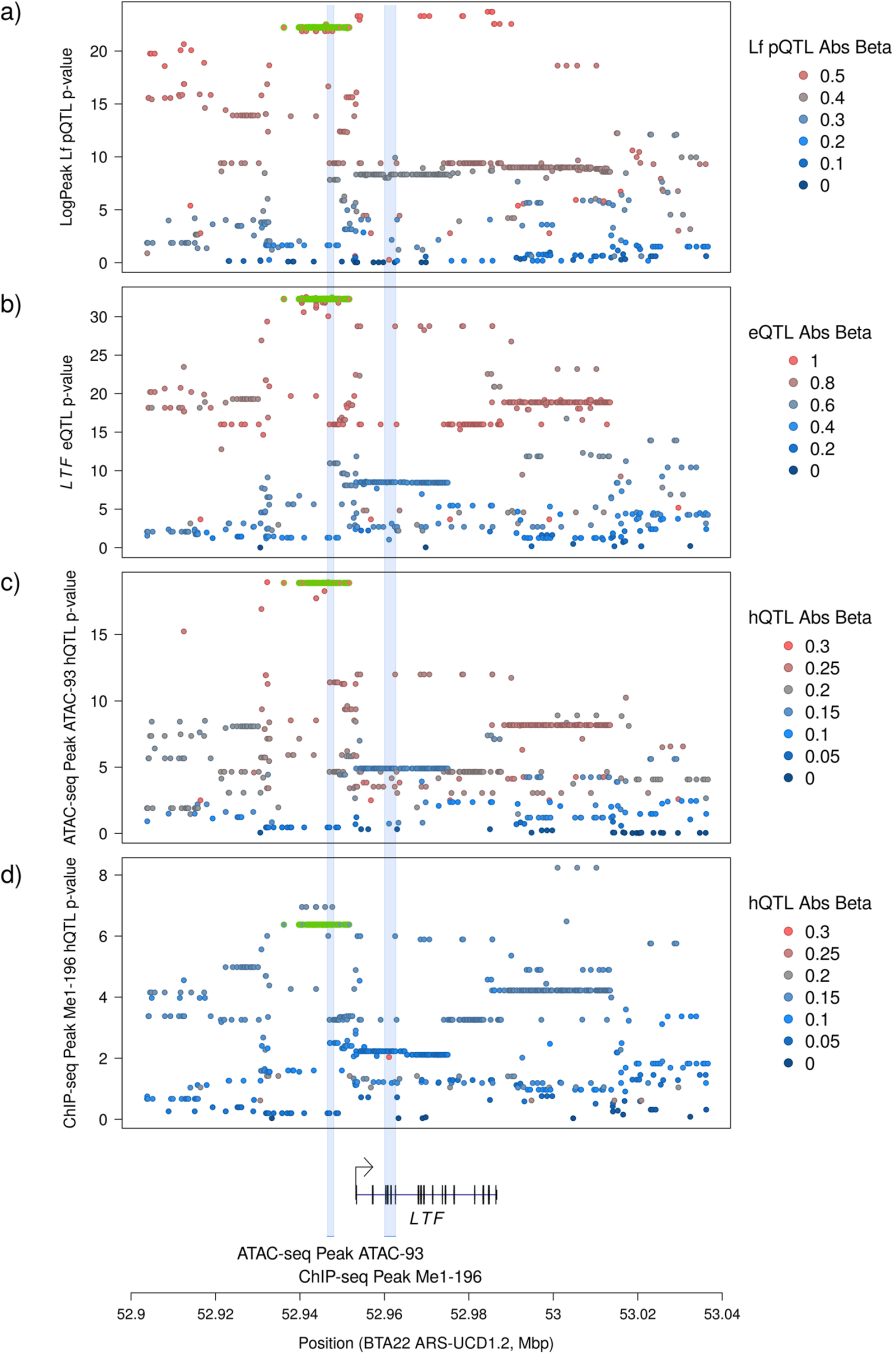
Fine mapping of the locus surrounding the *LTF* gene for Lf protein and co-located molecular QTL. **a**–**d** Show the sequence-resolution Manhattan plots of the QTL peaks for (**a**) the log-Peak Lf protein QTL; (**b**) the *LTF* eQTL; (**c**) the ATAC-seq caQTL for peak 93 (BTA22:52946445–52948019); and (**d**) the ChIP-seq hQTL for H3K4Me1 peak 196 (BTA22:52960026–52962680). All four are coloured by the absolute values of the association $\beta$-values. Variants highlighted in green are those which have absolute $\beta$-values in the top 5% for all four phenotypes. The positions of the *LTF* gene and the two chromatin peaks are indicated at the base of the figure

Shared genetic regulation underlying the hQTL, caQTL, *LTF* eQTL, and Lf pQTL is expected to be observed as correlations between both the allele effects of different QTL and the corresponding p-values. Moderate to strong correlations were observed between the *LTF* *cis*-eQTL and the pQTL identified for every Lf protein phenotype analysed (Table 4). These observations reinforce the assumption that differential Lf protein expression is under the same genetic control as differential *LTF* transcript abundance, and that the former likely derives from the latter. Strong correlations were also observed between the *LTF* *cis*-eQTL and several of the hQTL and caQTL (Table 5 and Additional file 1: Tables S14, S15). The strongest correlation between $\beta$ allele effects with the eQTL was seen for the ATAC-93 caQTL, and between $-\log _{10}(p)$-values for the Me1-196 hQTL. ATAC-93 sits upstream of the canonical *LTF* transcript, but overlaps the TSS of an alternative transcript (X1, XM_015459655). Peak Me1-196 overlaps four *LTF* exons (exons 3–6 in the coding sequence of both transcripts). We identified a set of 115 variants that fell within the top 5% (by absolute value of allele effect) of both hQTL, as well as the top 5% of both the *LTF* *cis*-eQTL and the LogPeak Lf pQTL. These variants are highlighted in green in Fig. 2, and will be labelled hereafter as the ‘core haplotype’ (see Additional file 1: Table S16 for a list of variants included in the haplotype). Of these variants, 15 map within ATAC-93, while none map within Me1-196. The LD between the top eQTL and LogPeak pQTL SNPs rs110395606 and rs133536129 was R^2^=0.842.

As 115 seemed a surprisingly large number of variants to be in such strong LD, we calculated the density of the variants surrounding the *LTF* locus in the imputation sequence population (excluding singleton variants), and compared this to the whole genome (see Additional file 4: Fig. S3). This analysis showed that the region immediately encompassing the core haplotype has a significantly higher density of variants than the genome as a whole (99.2 percentile).

Next, we examined whether SNPs in LD ($\text {R}^2 \ge 0.75$) with the top pQTL or eQTL SNPs fell within open chromatin regions. For the *LTF* eQTL, the top SNP was rs110395606 at position BTA22:52941483 ($p={1.504 \times 10^{-32}}$), which is in LD with 142 other variants. Of these variants, 107 fell within an open chromatin region: 42 in Me3-391, 26 in ATAC-93, 17 in ATAC-94, 14 in Me1-202, and 14 in Me1-196, with the remaining variants spread across another six regions. The majority of these regions also exhibited significant QTL that were strongly correlated with the eQTL (Table 5). A lower but still significant correlation was observed for Me3-391 (Pearson $r=0.759$ between the allele effects); however, this ChIP-seq region encompasses the ATAC-seq derived region ATAC-94, which gave a stronger correlation. For the LogPeak Lf pQTL (where the strongest correlations were observed with the eQTL; Table 4) the top associated SNP was rs133536129, at position BTA22:52984449. Using the same LD threshold of $\text {R}^2 \ge 0.75$ applied above, 165 variants were in strong LD, of which 87 mapped to an open chromatin region. Of these, 45 mapped to Me3-391 ($r=0.803$ between hQTL and pQTL), 22 to ATAC-93 ($r=0.913$), 17 to ATAC-94 ($r=0.907$), and 14 to Me1-196 ($r=0.908$), with the remaining six variants distributed across another five regions. The correlations observed between these chromatin QTL and the LogPeak pQTL were uniformly stronger than the equivalent correlations between the chromatin QTL and *LTF* eQTL.

**Fig. 3:**
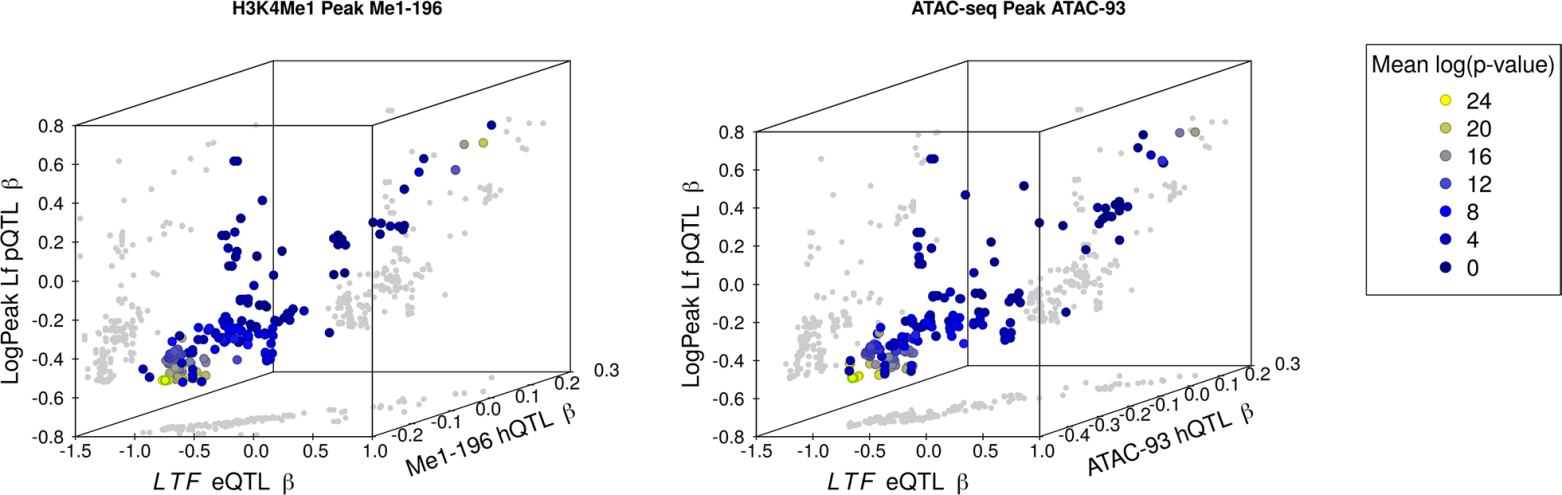
Three-dimensional scatter-plots of $\beta$-values for the lactoferrin pQTL and eQTL against two chromatin QTL. Left: the ChIP-seq H3K4Me1 peak 196 hQTL. Right: the ATAC-seq peak 93 caQTL. Both plots are coloured by the mean of the $\mathsf {\log _{10}}$-transformed p-values

After having established the correlations between pairs of QTL, we examined the three-way interactions among hQTL or caQTL, with eQTL and pQTL. Fig. 3 presents two three-dimensional scatter-plots of the $\beta$ allele effects, showing the relationships between LogPeak Lf, *LTF* gene expression, and chromatin accessibility for the two open chromatin regions Me1-196 and ATAC-93. The core haplotype variants are bordered with green as in Fig. 2, and appear as a single point because all 115 variants have very similar p-values across all traits. To summarise these two plots, and to facilitate comparisons between them, we performed a principal component analysis on each set of three allele effect variables. Then, we determined the percentage of variance for each set that could be explained by the first principal component, as a three-dimensional analogue to the R^2^ (a pseudo-R^2^). When calculated for the pQTL, eQTL, and Me1-196, this yielded 84.1%, and 87.4% with ATAC-93. Repeating this analysis using the $-\log _{10}(p)$-values instead of allele effects yielded 94.8% and 93.0%, respectively. Values obtained for other Lf phenotypes are provided in Table 6.

### Transcription factor binding site analysis

The co-occurrence of top-associated variants for both Lf protein and molecular phenotypes highlights a subset of candidates potentially causing these effects. To attempt to further differentiate these candidates, transcription factor binding site analysis was performed. Using a threshold for the minimum hQTL or caQTL p-value of ${7.9 \times 10^{-8}}$, 39 open chromatin regions with significant QTL were identified, comprising four for H3K4Me1 ChIP-seq, three for H3K4Me3 ChIP-seq, and 32 for ATAC-seq (see Additional file 1: Table S17). The sequence from these regions was extracted and analysed to predict TFBSs using TFBSTools and the JASPAR2020 database (see "Methods"). This identified 1285 TFBSs with a relative score $>0.9$ and a transcription factor matrix (TFM) p-value [44] $<{1 \times 10^{-5}}$. Of these 1285, the majority ($n=885$) were found within an ATAC-seq peak, followed by H3K4Me1 ChIP-seq windows ($n=270$), with the remainder ($n=130$) falling under three H3K4Me3 ChIP-seq peaks. The 1285 predicted sites included 80 under the Me1-196 peak (37 on the positive strand, matching the *LTF* gene), and 71 under the ATAC-93 peak (40 on the positive strand). In total, 1144 TFBSs were predicted from the CORE section of the JASPAR2020 database (see "Methods"), with the three most commonly predicted classes of transcription factors (TFs) being C2H2 zinc finger factors ($n=348$, from 37 open chromatin regions), basic helix–loop–helix factors ($n=170$, from 27 regions), and homeo domain factors ($n=112$, from 25 regions). The STAT family of TFs, which are known to affect the expression of lactation-related genes [3, 45], featured 46 predicted binding sites across 18 regions. Outside the CORE section of the database, the remaining 141 sites were identified in the POLII section, with the majority ($n=114$) being predicted downstream core elements (DCE).

Amongst the 115 core haplotype variants, five (all mapping within ATAC-93) were found within predicted TFBSs. Functionally, the most interesting predicted TF binding sites were found for rs110000337 (BTA22:52947400), which sat within sites for the two STAT domain containing TFs STAT5A/STAT5B (TFM $p={5.48 \times 10^{-6}}$) and STAT1 ($p={2.15 \times 10^{-6}}$), as well as a predicted site for the HMG domain containing TF SOX5A ($p<{1 \times 10^{-6}}$). On the negative strand, this SNP also overlapped with predicted binding sites for the TEA domain factors TEAD1 ($p={3.96 \times 10^{-6}}$) and TEAD2 ($p={3.38 \times 10^{-6}}$). Another SNP, rs137271649 (BTA22:52946852), sat within predicted binding sites for the homeo domain factor ZEB1 ($p={9.78 \times 10^{-6}}$) and the two basic helix–loop–helix factors TCF3 and TCF12 (both $p={3.34 \times 10^{-6}}$). SNPs rs109790251 (BTA22:52946581) and rs42013170 (BTA22:52946494) sat within predicted binding sites for the C2H2 zinc fingers ZNF148 (p-value $p={4.17 \times 10^{-6}}$) and ZNF423 ($p={4.2 \times 10^{-6}}$). SNP rs132785282 (BTA22:52947941) sat within a predicted binding site for NR2F1 ($p={6.24 \times 10^{-6}}$), which is another zinc finger containing TF. Including the remainder of the 107 eQTL variants that were in strong LD with the top variant, and mapped within an open chromatin region, resulted in one additional variant overlapping with a predicted TFBS. This variant, rs42013174 (BTA22:52946715, also within ATAC-93), co-located with predicted binding sites for the two fork head/winged helix TFs FOXA1 ($p={3.93 \times 10^{-6}}$) and FOXD1 ($p<{1 \times 10^{-6}}$).

## Discussion

### Regulatory QTL underlying lactoferrin concentration in milk

In this study, we have identified a number of QTL co-locating to the region surrounding the *LTF* gene. As expected, a strongly significant pQTL was found for Lf at all three sampling times. Additionally, an eQTL for *LTF* was also observed, and importantly, this showed a strong correlation with the pQTL (R^2^ up to 0.935; Table 4). This strong correlation implies that the two QTL are under shared genetic regulation, with the level of gene expression controlling the concentration of milk Lf protein. Furthermore, we have identified a number of open chromatin regions, some of which also present similar genetic signals to those underlying the eQTL and pQTL. This finding suggests the presence of a regulatory chain, with regulatory elements in open chromatin regions controlling gene expression, which in turn control milk protein concentration. Selecting variants that were strongly associated with the pQTL, eQTL, and the ATAC-93 and Me1-196 QTL identified a haplotype of 115 variants (the ‘core haplotype’), 15 of which are within ATAC-93. This open chromatin region is therefore a prime candidate region for the underlying causative variant(s). To facilitate this search, we examined the reference DNA sequence within this and other open chromatin regions surrounding the *LTF* gene to predict transcription factor binding sites that could affect milk protein expression. Variants mapping within these predicted binding sites were considered to be the best candidate causal variants controlling *LTF* expression, and thereby milk Lf concentration.

### Promoter-region variants

In this study, we identified a set of 115 variants (the core haplotype) that are strongly associated with milk Lf concentration, *LTF* gene expression, and chromatin openness in two regions near the *LTF* gene. Previous studies of the region upstream of the *LTF* gene have identified a number of variants. One set of 19 variants was identified by O’Halloran et al. [46]. Although no association analysis was performed with milk Lf concentrations or somatic cell score (SCS) in that work, eight of these variants fell within the core haplotype reported herein, and therefore were all strongly associated with Lf milk concentration in the current study, exhibiting p-values of ${5.87 \times 10^{-23}}$ for LogPeak and ${2.30 \times 10^{-32}}$ for LogLate. Beyond the core haplotype, another variant identified by O’Halloran et al. [46], “-28” (rs41256920) at BTA22:52953304, was directly adjacent to the annotated TATA box TFBS [47], and has been shown to be associated with milk Lf concentration in a small number of animals [40]. Four additional novel promoter variants were recently identified by Moncada-Laínez et al. [48] in Honduran dairy cattle; however, none of these variants were observed in our study population.

A small number of other genetic variants in the promoter region of the *LTF* gene have also been associated with Lf concentration in milk. One variant (rs43706485) in the 5′-UTR, frequently named “Lf+32” in the literature, has been characterised by several authors [39, 41], and is located at BTA22:52953364 in the current cattle reference (ARS-UCD1.2). These studies showed that Lf+32 is associated with both Lf concentration and SCS breeding value (BV; a marker of mastitis sensitivity), with the high Lf expression allele showing low SCS BV, and therefore increased resistance to mastitis. Within the current study, this variant is highly significant for both the LogPeak Lf phenotype ($p={8.08 \times 10^{-17}}$) and LogLate ($p={4.54 \times 10^{-20}}$), though these values are substantially less significant than those found for top associated variants in our study ($p={1.93 \times 10^{-24}}$ and ${1.06 \times 10^{-31}}$ for rs133536129, for example). Another promoter variant is “Lf-926” (rs135768375) at BTA22:52952404 [39]. Like Lf+32, Lf-926 has been associated with both SCS and Lf concentrations [49]. This variant has a similar significance level to Lf+32 in the current study, with p-values of ${2.32 \times 10^{-16}}$ and ${4.65 \times 10^{-21}}$ observed for the LogPeak and LogLate Lf phenotypes, respectively.

### Coding variants

Three missense mutations were observed in the study population; however, none of these were novel. All three variants were significantly associated with the LogLate Lf phenotype: Lys2Arg (rs384176726, BTA22:52953375) with $p={9.00 \times 10^{-8}}$, Ile145Val (rs52960814, BTA22:52960814) with $p={2.72 \times 10^{-13}}$, and His439Tyr (rs137554581, BTA22:52973728) with $p={7.09 \times 10^{-14}}$. The latter two variants were among the 47 variants that were reported in the *LTF* coding sequence by O’Halloran et al. [46], of which eight (including synonymous variants) were observed in the current study population. Amino acid positions are valid for both Ensembl protein sequence ENSBTAP00000001704 and RefSeq sequence XP_015315141.1, and include the 27 aa signal peptide. In an analysis comparing genetic signatures between measured and FT-MIR predicted traits that used some of the same data reported in the current study [50], the synonymous variant rs43765460 (Thr396 = BTA22:52969419) was proposed as being in strong LD with an unknown causative regulatory variant. This variant was very highly significant in the current study, with p-values of ${4.93 \times 10^{-24}}$ and ${2.47 \times 10^{-32}}$ for LogPeak and LogLate, respectively, and was also in strong LD ($\text {R}^2 > 0.85$) with the top variants for both LogPeak and LogLate. A large structural variant overlapping the *LTF* locus has been described within a French Holstein bull [51]. The 4.9-Mb inversion, annotated as esv3897783 in the Ensembl database, extends from BTA22:52320385 to BTA22:57234998 (remapped to the ARS-UCD1.2 reference genome), and overlaps 166 transcripts, including *LTF* (BTA22:52952571–52986619). However, its impact, if any, on *LTF* expression, or on Lf concentration in milk, is unknown.

### ChIP-seq and ATAC-seq

Due to the importance of chromatin state for regulating gene expression, we anticipated that hQTL and caQTL might be evident at the *LTF* locus, and that a subset of these would share genetic signals with the *LTF* *cis*-eQTL and Lf pQTL. As expected, several peaks exhibited such QTL, with Me1-196 and ATAC-93 giving the strongest correlations with the eQTL. The histone methylation peak Me1-196 mapped to coding exons 3–6, while the ATAC-seq peak mapped to a region adjacent to an alternative TSS for the *LTF* gene, and also overlapped with several candidate causal variants, in the core haplotype, for the regulation of Lf concentrations in milk. Interestingly, in spite of the core haplotype variants mapping near the alternative TSS, little to no expression of this alternative transcript can be seen in the mammary RNA-seq data set.

A second ATAC-seq peak, ATAC-94, also exhibited a caQTL that showed strong positive correlations with the *LTF* eQTL, and overlapped with the canonical *LTF* TSS, as well as with a predicted TATA box upstream [43]. Overlapping the same locus, the histone trimethylation peak Me3-391, which spans the window BTA22:52951823–52956227, gave an hQTL with a lower but still significant correlation (Pearson $\rho = 0.759$ for allele effects) with the eQTL. This broader peak also covered predicted binding sites for the TFs STAT3 and NF-$\kappa$B [43], in addition to the TATA box and canonical TSS. These TFs are involved in the regulation of mammary gland involution and the response to infection; both of these processes induce increased concentrations of Lf in milk.

### Transcription factors

We report three variants mapping to an ATAC-seq peak that are strongly associated with Lf phenotypes and sit within predicted TFBSs. In particular, the rs110000337 variant sits within predicted binding sites for several TFs with known effects on lactation and mammary gland development. Several of the STAT-domain TFs are important regulators of milk production and mammary gland status [52], and rs110000337 is predicted to alter binding sites for STAT5A, STAT5B, and STAT1. STAT5A and STAT5B fall within signalling pathways for at least two major galactopoietic hormones, prolactin and growth hormone [53], and have been shown to be involved in regulating the synthesis of both milk fat [54] and milk proteins such as $\alpha$S1-casein [55]. STAT1 shows different regulatory activities, but is important for the response to growth hormone during mammary gland growth and development [56], as well as for the response to bacteria during mastitis [57]: this latter function is particularly relevant to Lf as the protein has antibacterial properties. In addition to these three STAT-domain TFs, rs110000337 is predicted to alter a binding site for SOX5, a member of the SRY-related HMG-box family of TFs. SOX5 has several developmental functions, including cartilage formation [58] and influencing the migration of oligodendrocytes in the spinal cord [59], and SOX5 is also active in the terminal end bud of developing mammary glands [60], alongside the related TF SOX9. A second variant, rs132785282, was predicted to sit within the binding site for the nuclear hormone receptor NR2F1, also known as COUP-TF1. This acts as a co-receptor for the hormone oestradiol in mammary cells [61], and inducing its expression in a mammary cell line caused an increase in cell proliferation [62].

Previous studies have examined the sequence immediately upstream of the *LTF* gene to predict TFBSs [43, 63]. In our work, we identified a putative binding site for STAT5, as well as one for STAT3. STAT3 is a mediator of apoptosis and is upregulated at the time of mammary gland involution [60, 64] when Lf expression is also higher. None of the core haplotype variants highlighted in the current study fell within these binding sites. However, one core variant (rs134161490 at position BTA22:52949403) did map within a previously predicted binding site for AP-1 [43]. Like STAT3, the AP-1 family of TFs is induced in the mammary gland during involution [65]. Beyond the core haplotype, variant rs133094565 (BTA22:52950505) also fell within a predicted AP-1 binding site [43], and was moderately significant for associations with the LogPeak ($p={5.51 \times 10^{-7}}$) and LogLate ($p={6.56 \times 10^{-8}}$) phenotypes. Another important family of TFs is the nuclear factor NF-$\kappa$B family, which plays a major regulatory role in several aspects of immune function, and in upregulating the expression of STAT1 [66]. Two variants outside the core haplotype were found to map within predicted binding sites for the TF c-Rel [43], a member of the NF-$\kappa$B family. The first variant, rs381967837 (BTA22:52950613), gave p-values of ${4.19 \times 10^{-13}}$ and ${5.43 \times 10^{-17}}$ for LogPeak and LogLate, respectively. The second variant, rs135723142 (BTA22:52949971), gave less significant p-values of ${1.34 \times 10^{-6}}$ and ${6.56 \times 10^{-10}}$ for LogPeak and LogLate, respectively. A final significant variant, rs137735372 (BTA22:52951180), fell within a predicted GC Box element, and exhibited p-values of ${4.67 \times 10^{-13}}$ and ${2.15 \times 10^{-14}}$ for the LogPeak and LogLate phenotypes, respectively.

## Conclusions

The protein lactoferrin (Lf) is highly valuable for its iron-binding and antimicrobial properties. Using Lf protein concentrations in milk, we have identified a conspicuously large QTL, showing at least a doubling of secreted lactoferrin between opposing homozygous genotypes. To help elucidate the underlying genetic cause of this QTL, we have identified overlapping, correlated QTL for a range of additional omics data sets, comprising gene expression, histone modifications for several marks, and chromatin accessibility. Together, these analyses highlight a set of 115 variants (labelled herein the core haplotype) that are in the top 5% for all four of the protein, gene expression, and chromatin QTL. Among the core haplotype variants, several mapped within an open chromatin region (ATAC-93), and of these, three overlapped predicted TFBSs: rs109790251, rs110000337, and rs132785282. Of these three, rs110000337 appears the best overall candidate causative variant for the Lf QTL, on the basis that it interferes with predicted binding sites for the TFs STAT1, STAT5A, and STAT5B, all of which have well-described impacts on lactation phenotypes. Future experiments could test candidate variants for causality using techniques such as massively-parallel reporter assays (MPRA [67]) or hybridisation chain reaction fluorescence *in situ* hybridisation coupled with flow cytometry (HCR-FlowFISH [68]). Overall, these results present a genetic approach to identify high Lf-producing animals, which, when combined with specific management techniques, could be used to select animals with substantial increases in Lf production.

**Table 1:** Summary phenotypic and heritability statistics for Lf concentration

Period	NP	$\text {Mean} \pm \text {SD}$	Median	Skew	NG	$\mathsf {h^2_{\text {SNP}} \pm \text {SE}}$	LRT Pval
Peak	621	$\mathsf {98.3 \pm 88.2}$	70.0	2.37	595	$\mathsf {0.364 \pm 0.102}$	${3.21 \times 10^{-9}}$
LogPeak	621			− 0.09	595	$\mathsf {0.416 \pm 0.102}$	${1.34 \times 10^{-12}}$
Mid	648	$\mathsf {201.3 \pm 139.7}$	173.0	1.20	622	$\mathsf {0.151 \pm 0.081}$	${1.77 \times 10^{-4}}$
LogMid	648			− 0.94	622	$\mathsf {0.253 \pm 0.094}$	${1.66 \times 10^{-7}}$
Late	611	$\mathsf {186.5 \pm 113.8}$	163.0	1.53	588	$\mathsf {0.335 \pm 0.099}$	${3.14 \times 10^{-13}}$
LogLate	611			− 0.71	588	$\mathsf {0.413 \pm 0.103}$	${<1 \times 10^{-16}}$
Agg	700	$\mathsf {7.0 \pm 81.8}$	− 3.0	1.48	679	$\mathsf {0.433 \pm 0.091}$	${<1\times 10^{-16}}$

Concentrations are shown in units of mg/L. The Agg period represents the aggregated phenotype produced using a repeated-measures model in AS-REML. The Skew column contains the estimated sample skewness for each phenotype. The NP column shows the number of phenotyped animals recorded for each sampling period; the NG column is the number of animals both phenotyped and genotyped. The LRT Pval column shows the p-value for $H_0{:}h^2_{\text {SNP}}=0$, determined using a likelihood ratio test

**Table 2:** The top five most significant ChIP-seq and ATAC-seq peaks identified within 1 Mb of the *LTF* gene

Peak Name	Location (BTA22)	Score	Signal Value	Q-value	TSS
Ac-244	52541892–52549649	7113	64.64	711.34	*CCDC12*
Ac-592	53325802–53327345	3079	34.48	307.94	*FYCO1*^U^
Ac-418	52935669–52936535	2764	31.86	276.42	*LTF*^U^
Ac-630	53398218–53403461	2517	29.77	251.72	FYCO1^*^
Ac-64	52136724–52138123	2170	26.77	217.07	ELP6^*^
Me1-283	53332642–53338482	323	8.94	32.37	*FYCO1*
Me1-133	52560161–52586174	268	8.13	26.88	CCDC12^*^
Me1-129	52541850–52549226	265	8.02	26.55	*CCDC12*
Me1-367	53749632–53751815	239	7.66	23.90	LARS2^*^
Me1-194	52944846–52949322	239	7.72	23.90	*LTF*^U^
Me3-482	53325803–53327366	29773	178.25	2977.31	*FYCO1*^U^
Me3-106	52283328–52285089	29451	176.68	2945.13	*PTPN23*
Me3-567	53470756–53472052	26738	163.37	2673.81	*LZTFL1*
Me3-140	52360766–52363004	24267	151.06	2426.73	*KLHL18*,*KIF9*
Me3-164	52425038–52427859	22979	144.57	2297.99	–
ATAC-158	53625481–53627252	28987	13.14	2898.77	*SACM1L*
ATAC-49	52555035–52556223	22154	14.51	2215.48	CCDC12^*^
ATAC-145	53470617–53471757	18874	17.63	1887.42	*LZTFL1*
ATAC-128	53333362–53335224	17271	10.28	1727.13	*FYCO1*^U^
ATAC-54	52578451–52579389	16778	11.02	1677.87	CCDC12^*^

Results are shown for each of the three histone modifications, and ATAC-seq peaks within the same window. Peak names are allocated by the peak caller sequentially for each dataset. Peaks are ranked by Q-value ($-\log _{10}$-scale). Score, signal value, and q-value are as reported in the narrowPeak or broadPeak files created by the peak caller. The transcription start site (TSS) column lists the genes for which the TSS overlapped with the peak, or, for those marked with an asterisk, the gene within which the peak is located. A superscript U indicates that the peak maps within 20 kb upstream of the indicated gene

**Table 3:** Peaks from ChIP-seq and ATAC-seq that overlap with top pQTL SNPs for each Lf phenotype

Phenotype	Top SNP	Position	Peak name	Peak location	QVal
LogPeak	rs133536129	52984449	Ac-443	52984080–52984603	25.0
			Me1-202	52981402–52984701	10.2
			Me3-405	52984124–52984609	12.1
			ATAC-103	52984170–52984748	8.1
LogMid	rs384918755	52953612	Ac-425	52951712–52956226	166.6
			Me1-195	52950247–52958565	13.4
			Me3-391	52951824–52956227	952.3
			ATAC-94	52953062–52954626	443.6
LogLate	rs137774559	52946182	Ac-422	52944818–52948471	106.4
			Me1-194	52944846–52949322	23.9
			Me3-388	52945942–52946608	11.4
Agg	rs110659162	52986092	Ac-444	52985453–52986580	12.4
			Me1-203	52985484–52987687	3.4

Top SNP rsIDs and ARS-UCD1.2 positions (on BTA22) are given, along with overlapping peak locations on the same reference genome and chromosome. Peak names are given by dataset (histone modification type or ATAC-seq) and peak number as assigned by the peak caller. The QVal column represents the adjusted log-pvalue as calculated by the peak caller

**Table 4:** Correlations between Lf protein QTLs for several phenotype definitions, and the *LTF* eQTL

	$\beta$ allele affects		$-\log _{10}(p)$	
Phenotype	Pearson	Spearman	Pearson	Spearman
LogPeak	0.908	0.856	0.935	0.919
LogMid	0.846	0.719	0.873	0.845
LogLate	0.906	0.748	0.897	0.859
Agg	0.862	0.698	0.824	0.823

Correlations were calculated using variants (n=1467) within 100 kb of the TSS of the *LTF* gene, between the allele effects ($\beta$) and between the $\mathsf {-\log _{10}}$ p-values

**Table 5:** Correlations between the *LTF* eQTL and neighbouring ChIP-seq and ATAC-seq QTL within 1 Mbp of the *LTF* gene

Peak name	Location (BTA22)	$\beta$ allele affects		$-\log _{10}(p)$	
		Pearson	Spearman	Pearson	Spearman
Me1-196	52960027–52962680	0.834	0.851	0.966	0.969
Me1-202	52981401–52984701	0.781	0.735	0.848	0.813
Me3-213	52580146–52580284	− 0.688	− 0.788	0.892	0.855
ATAC-92	52940691–52941040	0.818	0.559	0.845	0.736
ATAC-93	52946446–52948019	0.909	0.833	0.908	0.883
ATAC-94	52953062–52954626	0.869	0.667	0.890	0.840
ATAC-113	53055448–53055807	− 0.631	− 0.619	0.854	0.815
ATAC-194	53809923–53810456	− 0.808	− 0.763	0.640	0.576

Both Pearson and Spearman (rank) correlations were calculated between both the QTL allele effects, and the log-scale p-values. Only hQTL and caQTL with a minimum $p<{1\times 10^{-5}}$ and an absolute correlation $\mathsf {>0.8}$ for at least one statistic are included. Correlations were calculated across markers within 50 kb of the *LTF* gene, i.e., between positions 52896110 and 53036647 on BTA22

**Table 6:** PCA pseudo-R^2^ values

	$\beta$ allele affects		$-\log _{10}(p)$	
Phenotype	Me1-196	ATAC-93	Me1-196	ATAC-93
LogPeak	0.841	0.874	0.948	0.930
LogMid	0.809	0.854	0.912	0.907
LogLate	0.831	0.870	0.924	0.921
Agg	0.772	0.819	0.880	0.882

Values show the percentage of variance explained by the first principal component, for datasets comprising the Log-Peak pQTL, *LTF* eQTL, the Me1-196 hQTL, and the ATAC-93 caQTL, and were calculated between the allele effects ($\beta$) and between the $\mathsf {-\log _{10}}$ p-values, across markers within 50 kb of the *LTF* gene, i.e., between positions 52896110 and 53036647 on BTA22

### Supplementary information

**Additional file 1:** **Table S1.** ChIP-seq H3K27ac peak locations (MACS). **Table S2.** ChIP-seq H3K4Me1 peak locations (MACS). **Table S3.** ChIP-seq H3K4Me3 peak locations (MACS). **Table S4.** ATAC-seq peak locations (MACS). **Table S5.** Lf pQTL (LogPeak) variant effects. **Table S6.** Lf pQTL (LogMid) variant effects. **Table S7.** Lf pQTL (LogLate) variant effects. **Table S8.** Lf pQTL (Aggregate) variant effects. **Table S9.** LTF eQTL variant effects. **Table S10.** ChIP-seq H3K27ac hQTL variant effects. **Table S11.** ChIP-seq H3K4Me1 hQTL variants effects. **Table S12.** ChIP-seq H3K4Me3 hQTL variant effects. **Table S13.** ATAC-seq caQTL variant effects. **Table S14.** Correlations between the LTF eQTL and hQTL/caQTL. **Table S15.** Correlations between the LogPeak pQTL and hQTL/caQTL. **Table S16.** Core haplotype variants and association statistics. **Table S17.** Predicted TFBSs under ChIP-seq/ATAC-seq peaks with significant hQTL/caQTL. 

**Additional file 2: Figure S1.** Predicted TFBSs under ChIP-seq/ATAC-seq peaks with significant hQTL/caQTL.

**Additional file 3:** **Figure S2.** Manhattan plots of GWAS for four Lf phenotypes using HD chip genotypes: the aggregate model phenotype, plus log concentrations measured during three time periods. Dashed red lines indicate the Bonferroni significance threshold of to 7.91 × 10^−8^.

**Additional file 4:** **Figure S3.** Density of variants surrounding the LTF gene region. Coloured lines indicate genome-wide 5 %, 25 %, 75 %, and 95% percentiles. Vertical grey lines indicate the position of the core haplotype.

## Data Availability

RNA-seq data are available on the Sequence Read Archive under project PRJNA682457. ChIP-seq data are available on the European Nucleotide Archive under project PRJEB52456.
