# Supplementary material for: A common regulatory haplotype doubles lactoferrin concentration in milk
Source: Genet Sel Evol. 2024 Mar 28;56:22. doi: 10.1186/s12711-024-00890-x (PMC11234695; doi:10.1186/s12711-024-00890-x)

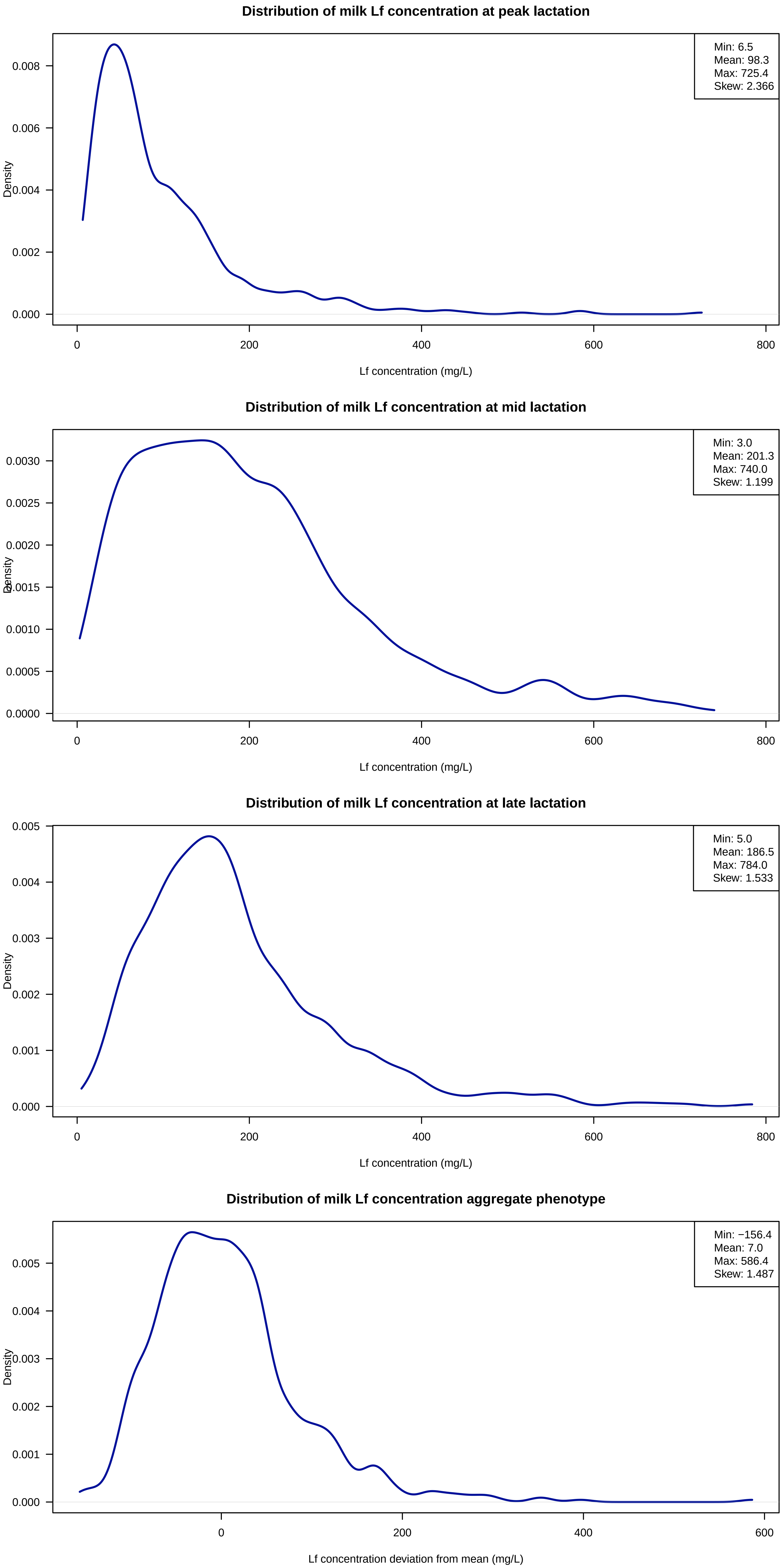
Distribution of milk Lf concentration at peak lactation
Min: 6.5
Mean: 98.3
Max: 725.4
Skew: 2.366
Density
0.000
0.002
0.004
0.006
0.008
0
200
400
600
800
Lf concentration (mg/L)
Distribution of milk Lf concentration at mid lactation
Min: 3.0
Mean: 201.3
Max: 740.0
Skew: 1.199
Density
0.0000
0.0005
0.0010
0.0015
0.0020
0.0025
0.0030
0
200
400
600
800
Lf concentration (mg/L)
Distribution of milk Lf concentration at late lactation
Min: 5.0
Mean: 186.5
Max: 784.0
Skew: 1.533
Density
0.000
0.001
0.002
0.003
0.004
0.005
0
200
400
600
800
Lf concentration (mg/L)
Distribution of milk Lf concentration aggregate phenotype
Min: −156.4
Mean: 7.0
Max: 586.4
Skew: 1.487
Density
0.000
0.001
0.002
0.003
0.004
0.005
0
200
400
600
Lf concentration deviation from mean (mg/L)

Distribution of log−scaled milk Lf concentration at peak lactation

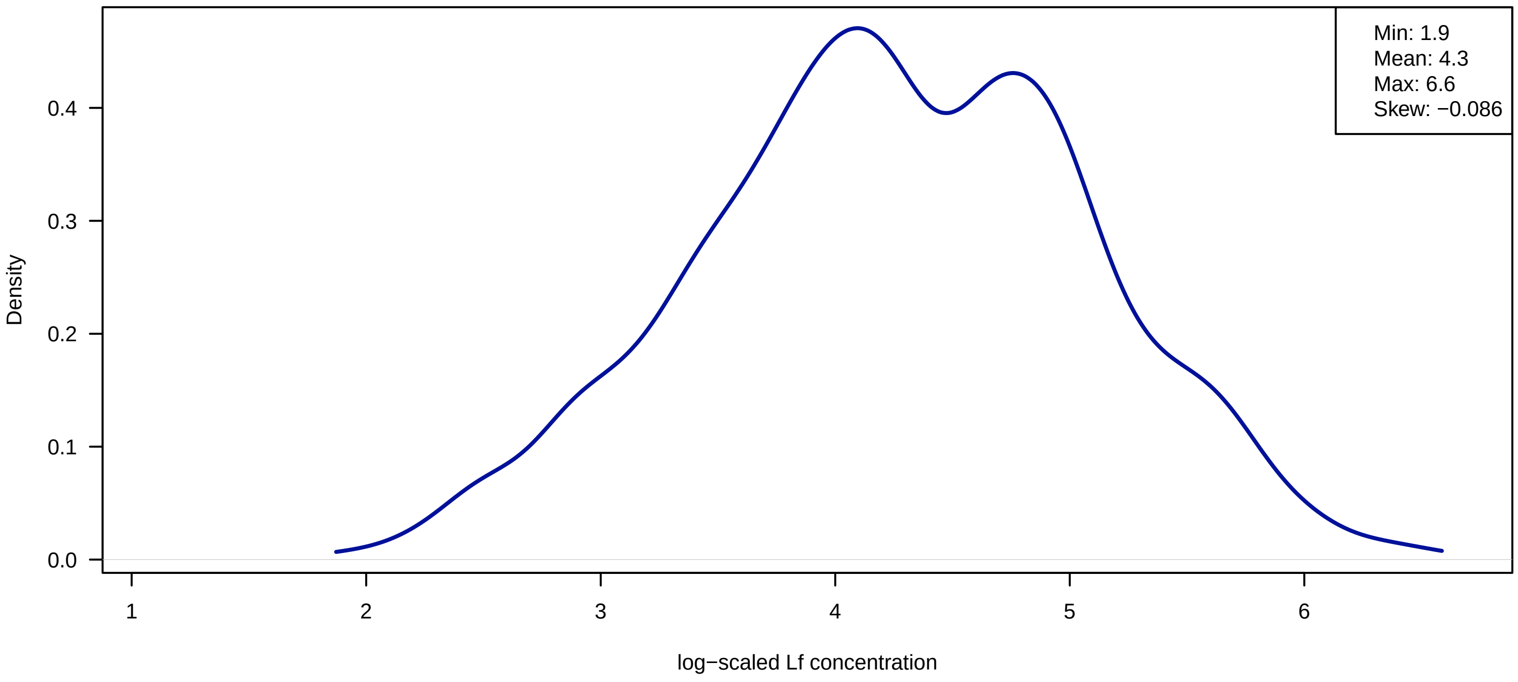

Distribution of log−scaled milk Lf concentration at mid lactation

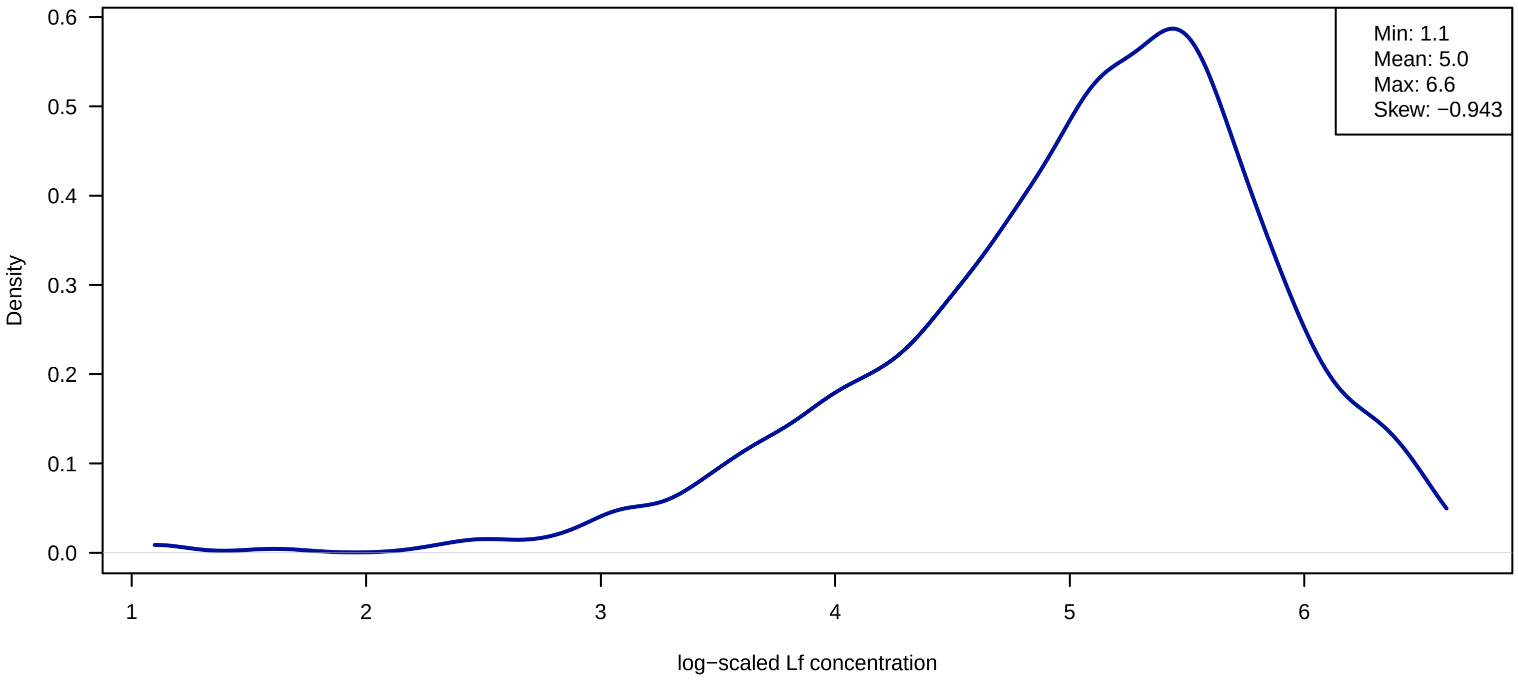

Distribution of log−scaled milk Lf concentration at late lactation

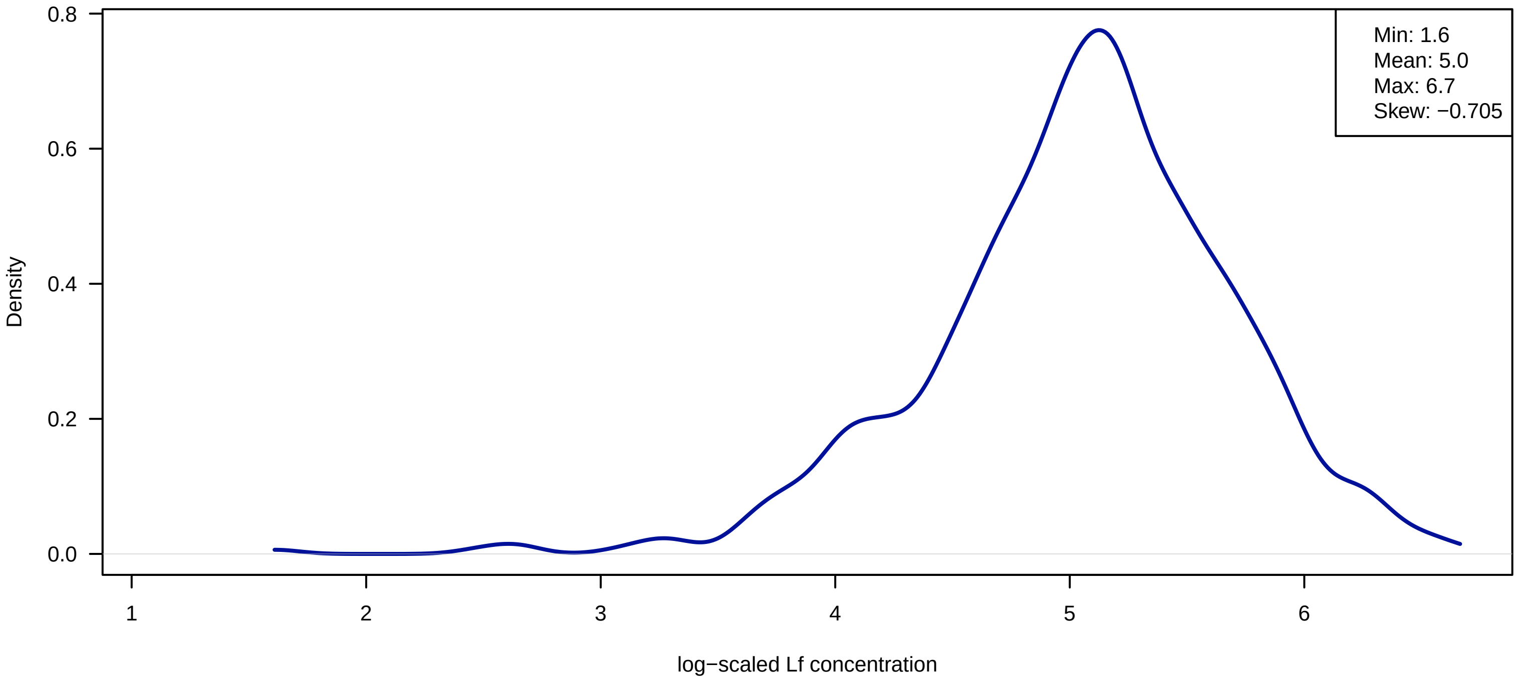

Supplement: Supplementary file 2 — Additional file 2: Figure S1. Predicted TFBSs under ChIP-seq/ATAC-seq peaks with significant hQTL/caQTL. [file 12711_2024_890_MOESM2_ESM.pdf]
