# Supplementary figures and images for: A common regulatory haplotype doubles lactoferrin concentration in milk
Source: Genet Sel Evol. 2024 Mar 28;56:22. doi: 10.1186/s12711-024-00890-x (PMC11234695; doi:10.1186/s12711-024-00890-x)

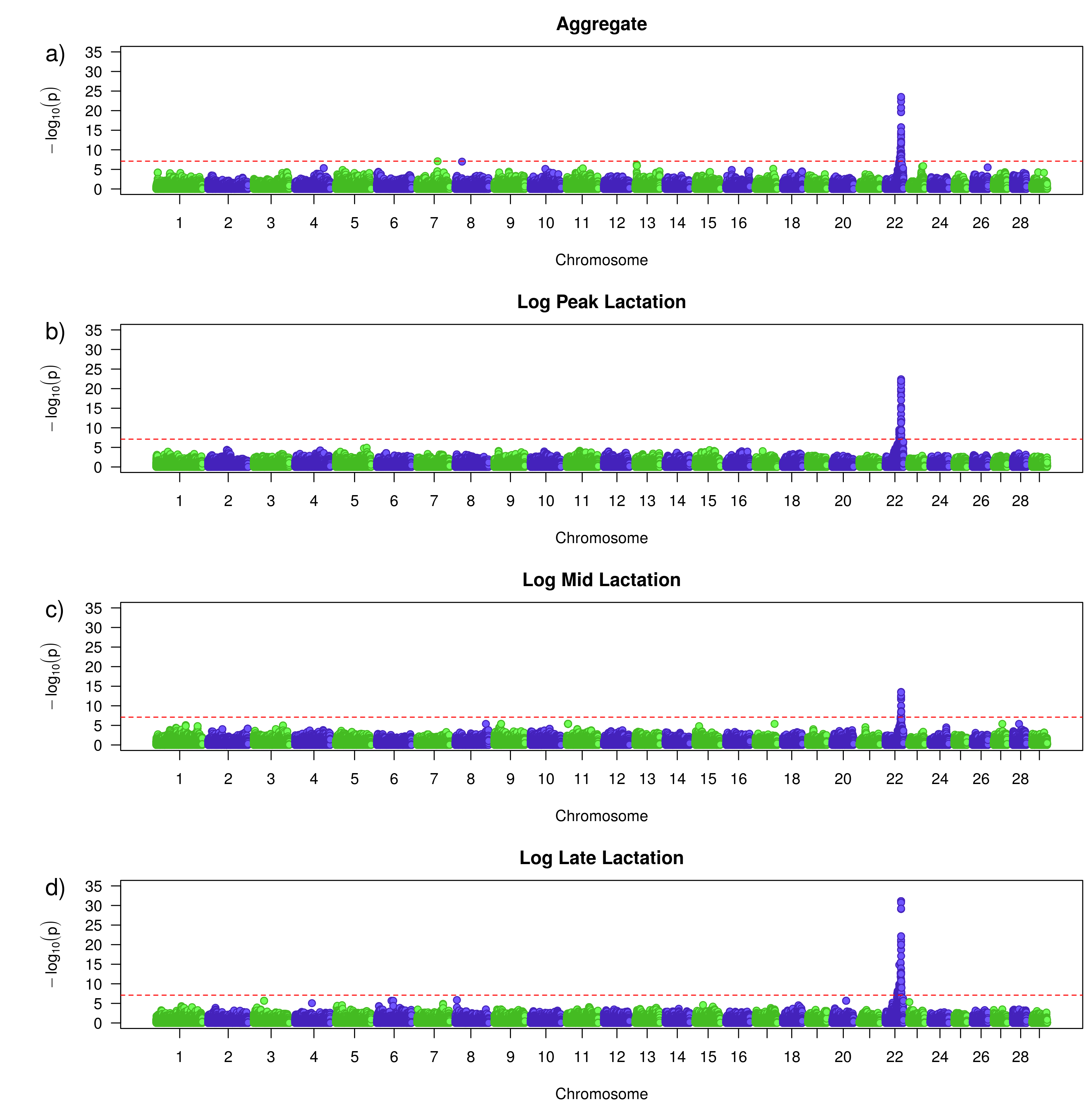

Supplement: Supplementary file 3 — Additional file 3: Figure S2. Manhattan plots of GWAS for four Lf phenotypes using HD chip genotypes: the aggregate model phenotype, plus log concentrations measured during three time periods. Dashed red lines indicate the Bonferroni significance threshold of to 7.91 × 10^−8^. [file 12711_2024_890_MOESM3_ESM.png]

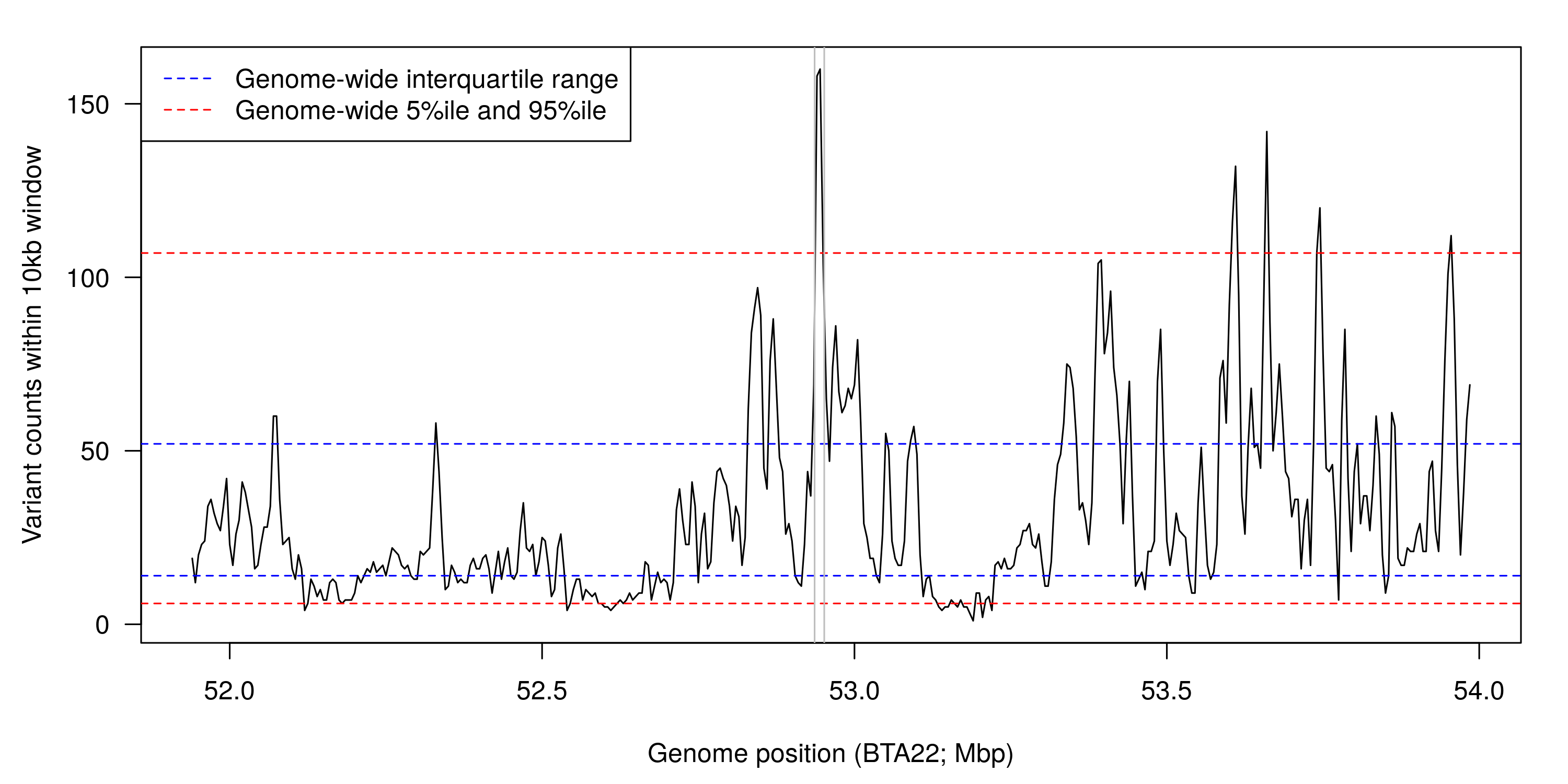

Supplement: Supplementary file 4 — Additional file 4: Figure S3. Density of variants surrounding the LTF gene region. Coloured lines indicate genome-wide 5 %, 25 %, 75 %, and 95% percentiles. Vertical grey lines indicate the position of the core haplotype. [file 12711_2024_890_MOESM4_ESM.png]
